# Host genetics, lung T-cell immunity, and laying activity determine the disease outcome in avian influenza virus-infected chickens

**DOI:** 10.1186/s13567-025-01689-4

**Published:** 2026-01-02

**Authors:** Luise Freier, Angele Breithaupt, Maryna Kuryshko, Diana I. Palme, Steffen Weigend, Elsayed M. Abdelwhab, Ulrike Blohm

**Affiliations:** 1 Institute of Immunology, Friedrich-Loeffler-Institut, Suedufer 10, 17493 Greifswald-Insel Riems, Germany; 2 Department of Experimental Animal Facilities and Biorisk Management, Friedrich-Loeffler-Institut, Suedufer 10, 17493 Greifswald-Insel Riems, Germany; 3 Institute of Molecular Virology and Cell Biology, Friedrich-Loeffler-Institut, Suedufer 10, 17493 Greifswald-Insel Riems, Germany; 4 Institute of Farm Animal Genetics, Friedrich-Loeffler-Institut, Hoeltystrasse 10, 31535 Neustadt, Germany; 5 Department of Infectious Diseases, Robert Koch Institut, Nordufer 20, 13353 Berlin, Germany

**Keywords:** Host genetics, laying activity, T-cell immunity, chicken, avian influenza virus

## Abstract

Avian influenza viruses pose a threat to both wild and domestic bird populations worldwide. In particular, chickens kept outdoors have contact with wild birds and thus an increased risk of infection with influenza viruses. Increasing use of high-performance chicken breeds in growing poultry production reduces genetic diversity whereas local breeds may offer valuable traits such as disease resilience important for sustainable agriculture. This study investigates the immunological performance of three local chicken breeds (Altsteirer, Ramelsloher, and Bielefelder) in Germany to assess their potential resilience to infectious diseases such as avian influenza. Viral resilience was assessed by inoculating 6-week-old chickens with a highly pathogenic avian influenza virus H7N1 and the moderately pathogenic recombinant avian influenza virus TG05-HA_R65_. Differences in mortality, immune response, transmission, virus shedding, and viral load in certain organs were observed. The Ramelsloher chickens had the lowest clinical score and the highest survival rate. Chicken-to-chicken transmission was only observed in the Altsteirer breed. Lung infiltration by T cells was detected in the Bielefelder and Ramelsloher breeds. In addition, 35-week-old laying hens at peak laying performance were infected with TG05-HA_R65_, to assess the impact of laying activity on viral resilience and immunocompetence. Compared with juvenile chickens, clinical signs, virus shedding, and seroconversion were detected at later time points. At the end of the experiment, most hens showed egg yolk peritonitis, likely caused by the influenza virus infection. This study indicates differences in the immunocompetence and viral resilience of local chicken breeds and provides breeding and biosafety management recommendations for organic farming.

**Supplementary Information:**

The online version contains supplementary material available at 10.1186/s13567-025-01689-4.

## Introduction

Chickens play an important role in global nutrition and agriculture, as the demand for poultry meat and eggs continues to grow [1]. At the same time, consumers and animal welfare advocates call for improved husbandry conditions, such as free-range systems used in organic farming [2]. However, these systems may increase infection risks through contacts with pathogens from wild birds [3, 4].

Wild birds serve as natural reservoirs for avian influenza viruses (AIV). Nevertheless, both wild birds and domestic poultry can be infected [5–8]. AIV are classified by their hemagglutinin (HA) and neuraminidase (NA) surface proteins, comprising 16 HA and 9 NA subtypes in birds [8–10]. Based on virulence and clinical outcomes, AIV are divided into highly-pathogenic (HPAIV) and low-pathogenic viruses (LPAIV) [6, 11, 12]. HPAIV (subtypes H5 or H7) carry a polybasic HA-cleavage site and cause acute, often fatal and systemic disease, while LPAIV infections typically result in mild or subclinical respiratory or gastrointestinal symptoms [13, 14]. The disease occurs globally and repeatedly affects poultry farms in Europe, Asia, and North America [15–17]. To control AIV, vaccination is permitted in some countries under strict conditions, but prohibited in Germany under the “Regulation on Protection against Avian Influenza” [18, 19].

Genetic diversity among chicken breeds contributes to differences in immunocompetence and disease resistance [20, 21]. Breed-dependent susceptibility to influenza viruses has been linked to T-cell activity and MHC-mediated mechanisms [22–25]. Despite the dominance of commercial high-performance chickens, local breeds may show greater resilience to infectious diseases [21, 26–28]. For example, a local Chinese chicken line exhibited lower mortality in a *Salmonella pullorum* challenge compared with a commercial chicken breed [26]. Nevertheless, other studies report higher susceptibility of local breeds to AIV infection [29, 30].

In a previous study, we analyzed immune cell composition in three local German chicken breeds (Altsteirer (ALT), Bielefelder (BIE), and Ramelsloher (RAM)) and found breed-specific differences under naive conditions [31]. The current study examines whether these differences affect immune performance following experimental infection with the model AIV TG05-HA_R65_ that has previously been used to determine immunological and clinical differences between different chicken lines [24].

As both immune function and egg production require high metabolic investment, reproductive activity may influence immunocompetence [32, 33]. In our previous work, naive laying hens showed no significant breed-specific differences in immune cell composition [31]. The present study therefore investigates how genetics, age, and laying performance affect the immune response to viral infection in the three local breeds ALT, BIE, and RAM.

## Materials and methods

### Cells and viruses

Madin-Darby canine kidney (MDCK-II) cells were obtained from the cell culture collection of the Friedrich-Loeffler-Institut, Riems, Germany. They were maintained in minimal essential medium (MEM) supplemented with Hank’s salts, Earle´s salts, sodium hydrogen carbonate (NaHCO_3_), nonessential amino acids (NEAA), sodium pyruvate, penicillin, streptomycin, and 10% fetal calf serum (FCS).

For this study, previously generated recombinant TG05-HA_R65_ was used for model virus infection in both juvenile chickens and laying hens: the LPAIV A/Teal/Germany/Wv632/2005 (TG05) carrying the HA and the polybasic HA-cleavage site from the HPAIV A/Swan/Germany/R65/06 (R65). An infection of 2-week-old SPF commercial-experimental White Leghorn chickens resulted in 30% mortality [14]. The reassortant virus was kindly provided by Jürgen Stech from Friedrich-Loeffler-Institut, Riems, Germany. HPAIV H7N1 A/Chicken/Italy/445/1999 was used for highly pathogenic infection in juvenile chickens. Infectious viral load was determined by plaque assay and plaque-forming units (PFU) on MDCK-II cells on 6-well plates.

### Animal experiments

The local breeds ALT, BIE, and RAM were generated from a parental population at the Institute of Agricultural Engineering at the University of Bonn, Germany and hatched at the Institute for Animal Welfare and Animal Husbandry of the Friedrich-Loeffler-Institut in Celle, Germany.

For the first experiment, day-old chicks (DOC) were transferred to the Friedrich-Loeffler-Institut in Greifswald, Insel Riems, Germany after hatching. They were raised in an approved biosafety level 2 (BSL2) facility and received *ad libitum* chick starter feed (PANTO® KAK Kükenalleinkorn, 2 mm pellets, PANTO Ecommerce GmbH, Hamburg, Germany) until the 5th week of age. From then on, the starter feed was mixed with a special rearing feed (PANTO® Unikorn, 3 mm pellets, PANTO Ecommerce GmbH, Hamburg, Germany) that the chickens were exclusively fed with from the beginning of the infection experiment. In order to guarantee adequate biosafety conditions, the chickens were transferred to a barn in the BSL3 facility of the Friedrich-Loeffler-Institut in Greifswald, Insel Riems, Germany, 1 week prior to the onset of the infection.

Ten 6-week-old chickens of each breed were inoculated oculonasally with 200 µl of either highly pathogenic H7N1 or moderately pathogenic TG05-HA_R65_ influenza virus solution containing 10^5^ PFU per animal. Five 6-week-old chickens of each breed were not inoculated and served as naive control animals. To assess viral transmission, five additional sentinel animals of the same breed and age were added 1 day post infection (dpi) to each group. Chickens were observed daily for 10 days for exhibiting any clinical signs and classified according to the standardized clinical scoring system by World Organization for Animal Health (WOAH) as healthy (0), ill (1), severely ill (2), or dead (3) [34]. Moribund animals were anesthetized by isoflurane inhalation and subsequently euthanized by opening the jugular vein and withdrawing blood. These animals were scored as dead on the following trial day. From each animal, 1 mL of blood was taken before the infection as well as 3 dpi and 7 dpi. Cloacal and oropharyngeal swab samples were taken into 1 mL swab sample medium (cell culture medium containing 1.0% Baytril, 0.5% Lincomycin, and 0.1% Gentamicin) at 2 dpi, 4 dpi, 7 dpi, and 10 dpi, shaken and then stored until further analysis at −80 °C. For necropsy at 4 dpi, three infected animals per group were anesthetized by isoflurane inhalation, subsequently euthanized in the same manner as described before, and organ samples were taken. Lung samples for flow cytometry analysis were collected in phosphate-buffered saline (PBS). Samples of brain, lung, spleen, and duodenum were taken and stored at −80 °C until further processing. Serum samples were collected from the surviving animals at 10 dpi and stored at −80 °C.

For the second experiment, laying hens were kept in floor pens until 34 weeks of age (4.8 animals per m^2^, 7 hens per nest, 18 cm perch per animal) by project partners at the Institute for Farm Animal Genetics of the Friedrich-Loeffler-Institut, Mariensee, Germany. The animals were fed *ad libitum* with feed for laying hens according to the guidelines of organic farming (Legefutter AF ÖTZ 80 TP, Meyerhof zu Bakum GmbH, Melle, Germany). At 34 weeks of age, the chickens were transferred to a barn in the BSL3 facility of the Friedrich-Loeffler-Institut in Greifswald, Insel Riems, Germany.

Ten 35-week-old laying hens of each breed were inoculated oculonasally with 200 µl of TG05-HA_R65_ virus solution containing 10^5^ PFU per animal. Owing to limited animal availability, only a single noninoculated control animal could be included in the BIE group, whereas five naive control animals were used in the other two groups. The chickens were observed daily for 10 days for exhibiting any clinical signs as described before. Blood, swab, and organ samples were collected in the same manner as in the trial with the 6-week-old chickens.

### RNA extraction

To determine viral load, viral RNA was extracted from organ samples taken at necropsy at 4 dpi. To each sample, a 5 mm steel bead and 1.0 mL of cell culture medium were added. Subsequently, samples were homogenized in a TissueLyzer II (QIAGEN, Hilden, Germany) and RNA was extracted by the KingFisher™ Flex System (Thermo Fisher Scientific, Waltham, MA, USA) using KingFisher^®^ 96 Accessory Kit A and NucleoMag^®^ VET Kit (MACHEREY NAGEL GmbH & Co. KG, Düren, Germany) according to the manufacturer’s instructions.

To analyze virus shedding, viral RNA from swab samples was extracted in an analogous manner using the same kits as mentioned before.

### RT-PCR

One-step RT-PCR was used to detect the influenza A virus (IAV) genome M1.2 and determine the quantification cycle (Cq) in organ and swab samples. The IAV-M1.2.Mix-FAM primer-probe mixes utilized were modified in accordance with the publication by Spackman et al. [35]. The pan-influenza A real-time RT-PCR assay was combined with a heterologous internal control system (Table 1) [36].

**Table 1:** **Oligonucleotides used in this study**

Oligo	Sequence (5′-3′)
IAV-M1-F	AGA TGA GTC TTC TAA CCG AGG TCG
IAV-M1.1-R	TGC AAA AAC ATC TTC AAG TYT CTG
IAV-M1.2-R	TGC AAA GAC ACT TTC CAG TCT CTG
IAV-M1-FAM	FAM-TCA GGC CCC CTC AAA GCC GA-BHQ1
EGFP-11-F	CAG CCA CAA CGT CTA TAT CAT G
EGFP-10-R	CTT GTA CAG CTC GTC CAT GC
EGFP-1HEX	HEX-AGC ACC CAG TCC GCC CTG AGC A-BHQ1

IAV-M1.2-Mix-FAM modified from [35] and EGFP-Mix4 (5)-HEX [36]

One-step RT-PCR was carried out using the AgPath-ID™ One-Step RT-PCR Kit (Applied Biosystems, Thermo Fisher Scientific, Waltham, MA, USA) according to the manufacturer’s instructions. The reactions were run on a CFX 96-ORM Real-Time PCR System (Bio-Rad Laboratories, Hercules, CA, USA) using the following thermal profile: 10 min at 45 °C for reverse transcription, followed by an initial activation step of 10 min at 95 °C. Amplification was performed over 42 cycles of 95 °C for 15 s (denaturation), 55 °C for 20 s (annealing, with fluorescence acquisition), and 72 °C for 30 s (elongation). The cut-off was set at a Cq value of 38.

### Seroconversion

To investigate seroconversion, serum samples were collected at 10 dpi at the end of the trial and stored at −80 °C until further processing. They were then inactivated (56 °C, 120 min) and analyzed by a commercially available competitive enzyme-linked immunosorbent assay (ELISA) for the detection of antibodies to IAV nucleoprotein (NP) according to the manufacturer’s instructions (ID Screen Influenza A Antibody Competition FLUACA, Innovative Diagnostics, Grabels, France). Optical density (OD) was measured by a Spark^®^ Multimode Microplate Reader (Tecan Group Ltd., Männedorf, Switzerland) and results with a sample-to-positive-rate (S/P) equal or lower than 45% were considered as positive by the manufacturer’s guidelines. Samples with an S/P rate higher than 50% were defined as negative, samples between 45 and 50% as questionable.

### Isolation and cell staining of leukocytes from blood and lung samples for flow cytometry analysis

To evaluate the cellular immune response to viral infection, blood and lung samples were analyzed by flow cytometry. Peripheral blood mononuclear cells (PBMC) from blood samples were isolated by density centrifugation over a Pancoll-gradient (Pancoll human, Density: 1.077 g/mL, PAN-Biotech, Aidenbach, Germany). After centrifugation for 30 min at 760 × *g* and room temperature, 1.0 mL of plasma was separated and stored at −70 °C until further use. Lung samples were collected at necropsy and kept in PBS until they were immediately processed in the laboratory. The lungs were minced into 23 mm pieces and 1.0 mL of digestion mix containing serum-free cell culture medium, Collagenase IV (2.0 mg/mL, Biochrom, Berlin, Germany) and Type IV DNase I (0.1 mg/mL, Sigma-Aldrich Chemie GmbH, Taufkirchen, Germany) was added to each sample. After incubation for 1 h at 37 °C and 1200 rpm, the tissue and debris were removed by filtration and the samples were washed. After the isolation process, lung leukocytes as well as PBMC were stained for flow cytometry analysis (Table 2) and measured by the BD LSRFortessa™ cell analyzer (BD Biosciences, San Jose, CA, USA). Compensation and interpretation were done using BD FACSDIVA™ software and FlowJo.

**Table 2:** **Cell staining for flow cytometry analysis of immune cell composition**

Primary antibodies				
Antigen	Clone	Conjugate	Manufacturer	Concentration
Chicken CD25	AV142	Pure	Bio-Rad AbD serotec	1.00 µg/mL
Chicken CD8β	EP42	Pure	Southern Biotech	0.83 µg/mL
Chicken CD4	2–35	Pure	serotec	1.00 µg/mL
Aqua zombie		V500	Biolegend	ATM^a^
Chicken γδ TCR	TCR-1	FITC	Southern Biotech	5.00 µg/mL
Chicken CD41/CD61	11C3	PE	Bio-Rad AbD serotec	ATM^a^
Chicken Bu-1	AV20	Alexa Fluor 647	Southern Biotech	0.50 µg/mL
Chicken CD3	CT-3	Pacific blue	Southern Biotech	5.00 µg/mL
Chicken CD8α	CT-8	Biotin	Southern Biotech	5.00 µg/mL

Secondary antibodies				
Antigen	Clone	Conjugate	Manufacturer	Concentration
Mouse IgG1	RMG1-1	BV650	Biolegend	4.00 µg/mL
Mouse IgG2a		PerCP	dianova	5.00 µg/mL
Mouse IgG2b		PE-Cy7	Southern Biotech	0.63 µg/mL
STAV / Streptavidin		BV711	Biolegend	0.50 µg/mL

^a^According to manufacturer’s instructions

### Histopathology and immunohistochemistry

Full autopsy was performed on all animals under BSL3 conditions. Samples of TG05-HA_R65_-infected RAM, ALT, and BIE layer hens (*n* = 3 per group) and noninfected controls (ALT: *n* = 5, RAM: *n* = 5, BIE: *n* = 1) were taken, including lung, spleen, brain, magnum, shell gland, and ovary. Following immersion-fixation in 10% neutral buffered formalin, tissues were paraffin-embedded and 2–4 μm-thick sections were stained with hematoxylin and eosin (HE). Immunohistochemistry (IHC) was performed for viral antigen detection using a primary antibody against the M protein of IAV (ATCC clone HB-64) as described [37]. Slides were scanned using a Hamamatsu S60 scanner and analyzed using NDPview.2 plus software (Version 2.8.24, Hamamatsu Photonics, K.K. Japan). HE-stained sections were evaluated, described and graded as scores 0 = no lesion, 1 = minimal/oligofocal, 2 = mild/multifocal, 3 = moderate/coalescing, 4 = severe/diffuse lesions. Following IHC, the distribution of IAV matrix protein was recorded on an ordinal scoring scale with scores 0 = no antigen, 1 = oligofocal, affected cells/tissue < 5% or up to 3 foci per tissue; 2 = multifocal, 6%–40% affected; 3 = coalescing, 41%–80% affected; 4 = diffuse, > 80% affected. Evaluation and interpretation were performed by a trained veterinarian (L.F.) and reviewed by a board-certified (DiplECVP) pathologist (A.B.) in a blinded fashion.

### Statistical analysis

All statistical analyses were performed using GraphPad Prism version 10.4.0 (GraphPad Software, San Diego, CA, USA). Statistical significance was defined at an alpha level of 0.05 (*P* < 0.05). Significance levels were indicated as follows: *P* < 0.05 (*), *P* < 0.01 (**), and *P* < 0.001 (***).

Ordinary one-way analysis of variance (ANOVA) with Šídák’s multiple comparisons test was used to analyze immune cell composition in all lung samples and in blood samples of H7N1-infected juvenile chickens. Normality and variance homogeneity were assessed using Brown-Forsythe and Bartlett’s tests. Two-way ANOVA with Tukey’s multiple comparisons test was used to analyze viral load in organs, oropharyngeal and cloacal viral shedding of sentinel animals. Mixed-effects analyses with Tukey’s multiple comparisons test were used to analyze oropharyngeal and cloacal viral shedding of inoculated animals and immune cell compositions in blood samples of all TG05-HA_R65_ infected chickens.

## Results

### Host genetic background and viral pathogenicity shape resilience to AIV infection

**Figure 1:**
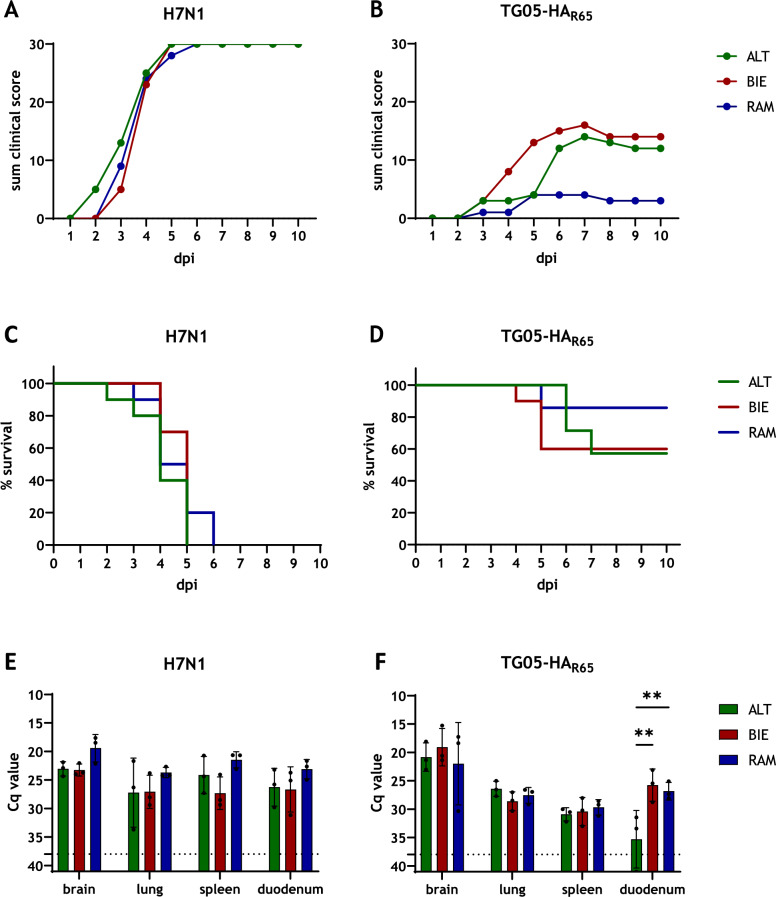
**Breed-specific differences of 6-week-old chickens following avian influenza infection.** Three different chicken breeds were infected with either highly pathogenic H7N1 (**A**) or moderately pathogenic TG05-HA_R65_ (**B**) and scored daily for 10 days as healthy (0), ill (1), severe ill (2), or dead (3), shown is the sum of the clinical score. Kaplan-Meier survival curves of chickens infected with H7N1 (**C**) or TG05-HA_R65_ (**D**) indicate the mortality rates as percent survival. Viral load in organ samples taken during necropsy at 4 dpi was assessed by RT-PCR for H7N1-infected chickens (**E**) or TG05-HA_R65_-infected chickens (**F**). The dotted line marks the Cq threshold value of 38. Data are shown as mean with standard deviation. Asterisks indicate statistical significance: (**) *P* < 0.01. ALT: Altsteirer, BIE: Bielefelder, RAM: Ramelsloher, dpi: days post-infection

To assess breed-specific susceptibility to viral pathogens, 6-week-old chickens were infected either with highly pathogenic H7N1 or moderately pathogenic TG05-HA_R65_. During H7N1 infection, no-to-minor differences in clinical signs and kinetics of the disease between the breeds were observed and all animals died from infection. First clinical symptoms occurred 2 dpi in the ALT group (Figure 1A). In the RAM group, the animals survived the longest until 6 dpi, while in the other two groups, all animals were dead by 5 dpi (Figure 1C). In contrast, greater differences between the breeds were observed during TG05-HA_R65_ infection (Figures 1B, D). Here, the RAM chickens had the lowest clinical score and the lowest mortality rate, with only one individual dying during the trial (mortality (RAM) = 14%). BIE chickens had the highest clinical score and they showed the strongest increase in clinical symptoms. From 7 to 10 dpi, differences between the ALT and the BIE groups were found to be minor. Both groups had comparable mortality rates (mortality (ALT) = 43%, mortality (BIE) = 40%).

To compare viral loads in the organs, samples of the brain, lungs, spleen, and duodenum were collected during necropsy at 4 dpi and analyzed by RT-PCR (Figures 1E, F). In H7N1 infected chickens, no significant differences between the breeds could be detected. Overall, RAM chickens had the highest viral load in all organs. In TG05-HA_R65_-infected chickens, the lowest viral load was found in the duodenum of ALT chickens (mean Cq value (ALT) = 35.3, mean Cq value (BIE) = 25.8, mean Cq value (RAM) = 26.8). The highest viral load of TG05-HA_R65_ was found among all breeds in the brain (mean Cq value (ALT) = 20.8, mean Cq value (BIE) = 19.1, mean Cq value (RAM) = 22.0).

### Host genetics determine lung infiltration by terminally differentiated CD8+ γδ T cells and cytotoxic CD8α+ αβ T cells in AIV infected chickens.

**Figure 2:**
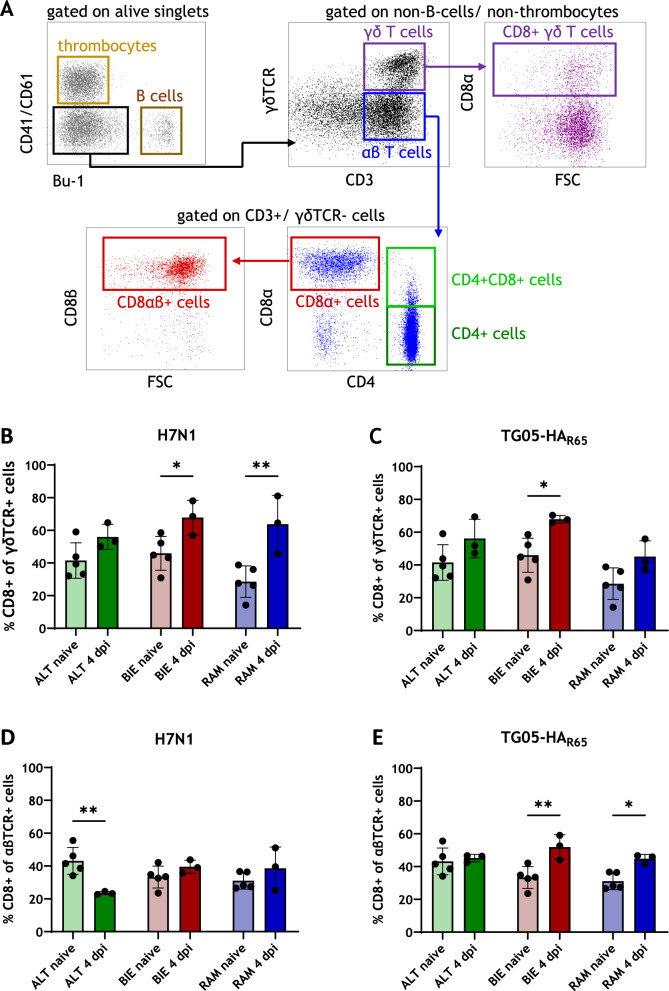
**Lung infiltration by T cells at 4 dpi.** Flow cytometry gating scheme for analysis of T cell subpopulations (**A**). CD8+ γδ T cells (**B**) and CD8+ αβ T cells (**D**) of H7N1-infected chickens compared with noninfected control animals. CD8+ γδ T cells (**C**) and CD8+ αβ T cells (**E**) of TG05-HA_R65_-infected chickens compared with noninfected control animals. Data are shown as mean with standard deviation. Asterisks indicate statistical significance: (*) *P* < 0.05, (**) *P* < 0.01. ALT: Altsteirer, BIE: Bielefelder, RAM: Ramelsloher, dpi: days post-infection

To investigate whether different breeds respond differently to viral infection, PBMC and lymphocytes derived from lung samples were examined by flow cytometry at 4 dpi. Figure 2A shows the gating scheme for leukocyte subpopulations. In the 6-week-old animals that were infected with TG05-HA_R65_, an increase in CD8+ γδ T cell receptor (TCR) + T cells in the lung was observed among all breeds at 4 dpi (Figure 2C). The strongest infiltration of CD8+ γδ T cell in the lung was found in the BIE breed. In both, the ALT and RAM breeds, this increase existed; however, it was not significant. In contrast, an increase in CD8α+ αβ TCR+ cytotoxic T cells was only observed in the BIE and in the RAM breeds (Figure 2E). In the ALT breed, the proportion of cytotoxic T cells did not differ between naive and infected animals at 4 dpi. In animals infected with the highly pathogenic H7N1 virus, a lung infiltration by CD8+ γδ TCR+ T cells was observed across all breeds at 4 dpi, comparable to the response seen in TG05-HA_R65_-infected animals. Here, this increase was the strongest in the RAM breed, followed by the BIE breed (Figure 2B). A slight, though not statistically significant, increase in CD8α+ αβ TCR+ cytotoxic T cells was also observed in the BIE and in the RAM breeds, albeit to a limited extent (Figure 2D). Notably, the ALT breed exhibited a marked decrease of these classical cytotoxic T cells at 4 dpi.

Other changes in the proportions of T cell subpopulations in the lung and in the blood constantly showed no breed-specific differences (Additional files 1 and 2).

### Virus transmission is restricted to ALT chickens infected with TG05-HA_R65_

**Figure 3:**
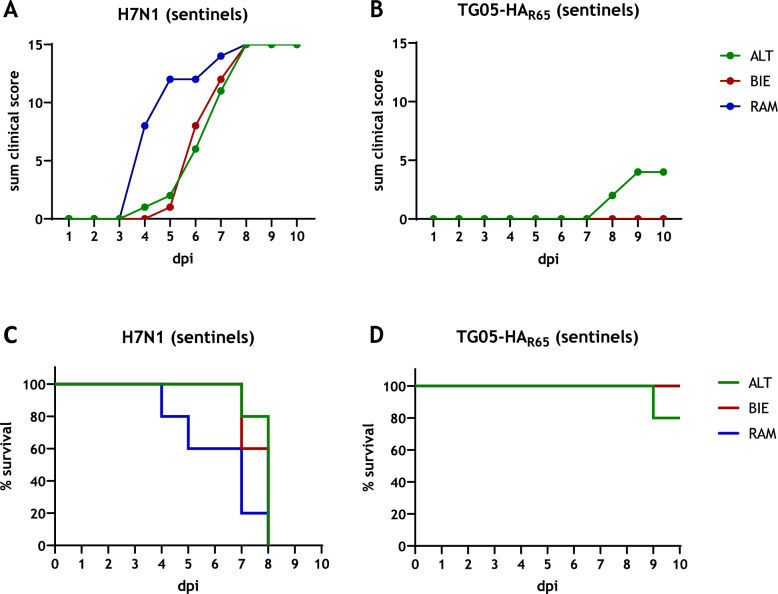
**Virus transmission.** At 1 dpi, five sentinel animals were put to each group of either H7N1-infected chickens (**A**) or moderately pathogenic TG05-HA_R65_-infected chickens (**B**) and scored daily for 10 days as healthy (0), ill (1), severe ill (2), or dead (3), shown is the sum of the clinical score. Kaplan-Meier survival curves of sentinel animals in the H7N1 infection groups (**C**) or TG05-HA_R65_ infection groups (**D**) indicate the mortality rates as percent survival. ALT: Altsteirer, BIE: Bielefelder, RAM: Ramelsloher, dpi: days post infection

To analyze whether there are breed-specific differences in virus transmission, sentinels were introduced 1 dpi and scored daily for exhibiting clinical symptoms. In the groups that received highly pathogenic H7N1, all sentinels got sick and died until 8 dpi. While differences between H7N1 infected ALT and BIE animals were found to be minor, RAM sentinels became ill and died the fastest (Figures 3A, C). In contrast, in the groups that were infected with TG05-HA_R65_, only two ALT sentinels showed clinical symptoms and one of them died (Figures 3B, D).

**Figure 4:**
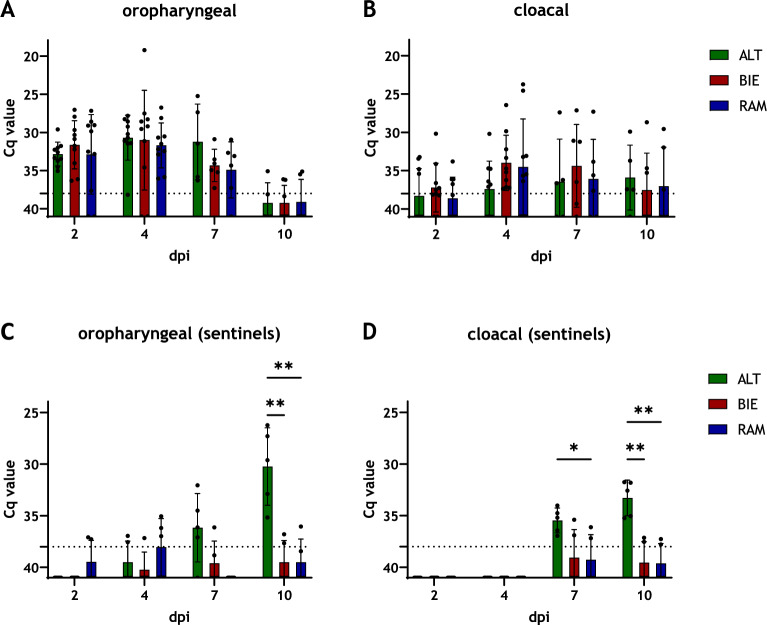
**Virus shedding in the TG05-HA**_**R65**_ infection groups. Oropharyngeal (**A**) and cloacal (**B**) virus shedding of TG05-HA_R65_-infected chickens were analyzed by RT-PCR at 2, 4, 7, and 10 dpi. Oropharyngeal (**C**) and cloacal (**D**) swab samples of sentinel animals were analyzed in the same manner. The dotted line marks the Cq threshold value of 38. All data are shown as mean with standard deviation. Asterisks indicate statistical significance: (*) *P* < 0.05, (**) *P* < 0.01. ALT: Altsteirer, BIE: Bielefelder, RAM: Ramelsloher, dpi: days post-infection

To explain the differences in virus transmission in the TG05-HA_R65_ experiment, oropharyngeal and cloacal swab samples were analyzed by RT-PCR for virus shedding. ALT chickens shed oropharyngeally more virus particles at 7 dpi compared with both BIE and RAM chickens (mean Cq value (ALT) = 31.2, mean Cq value (BIE) = 34.3, mean Cq value (RAM) = 34.9). Moreover, the viral load in swab samples of ALT chickens stayed at a comparable level on that day compared with the previous time point of examination at 4 dpi, while the virus shedding in BIE and RAM chickens already decreased (mean Cq value (ALT) = 30.7, mean Cq value (BIE) = 31.0, mean Cq value (RAM) = 31.7). At the end of the trial, independently of breed, all surviving animals showed a remarkable reduction in oropharyngeal virus shedding (Figure 4A), indicating clearance of the virus. In contrast, at 4 dpi, cloacal swab samples of ALT chickens indicated a tendency toward lower virus shedding compared with BIE and RAM chickens (mean Cq value (ALT) = 37.4, mean Cq value (BIE) = 34.0, mean Cq value (RAM) = 34.5). However, the viral load in cloacal swab samples remained at comparable levels across all breeds in subsequent examinations (Figure 4B). In this experiment, all animals were confirmed to be infected, as demonstrated by either the presence of viral RNA detected in swab samples or by seroconversion (Figures 4A, B, Additional file 3A). Interestingly, ALT sentinels shed significantly the most virus both cloacally and oropharyngeally. The two ALT individuals that shed the most virus oropharyngeally were tested positive for antibodies against NP of IAV. In contrast, no notable levels of virus particles were detected in swab samples of BIE and RAM sentinels (Figures 4C, D) and none of them seroconverted (Additional file 3A).

### Laying performance influences disease dynamics and clinical outcomes following AIV infection

To examine the influence of age and in particular laying performance on immunocompetence, IAV seronegative laying hens at their peak performance were infected with moderately pathogenic TG05-HA_R65_. The model virus was selected for this experiment as it facilitates the assessment of genetically determined variations in disease outcomes, as evidenced by the experiment conducted on juvenile chickens. Interestingly, in this experiment, there were individuals that neither seroconverted nor tested AIV positive by RT-PCR analysis of the swab samples (ALT: *n* = 1; BIE: *n* = 1), and thus, may possibly not have been infected. These animals were excluded from all analyses and figures. Furthermore, in contrast to the infected juvenile chickens, the majority of laying hens did not develop antibodies to NP of IAV at 7 dpi. Moreover, three surviving chickens had no antibodies to NP until the end of the trial at 10 dpi. However, these individuals were tested positive for virus shedding by RT-PCR.

**Figure 5:**
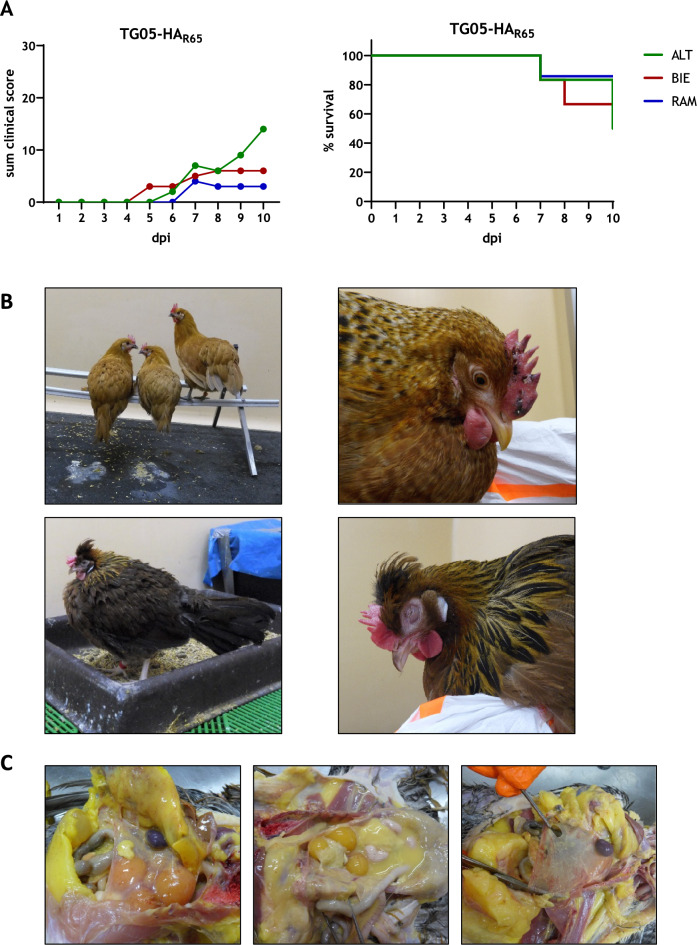
**Observations during TG05-HA**_**R65**_ infection of 35-week-old laying hens. The chicken breeds were infected with moderately pathogenic TG05-HA_R65_ at their peak laying performance and scored daily for 10 days as healthy (0), ill (1), severe ill (2), or dead (3), shown is the sum of the clinical score (**A**, left). Kaplan-Meier survival curves indicate the mortality rates as percent survival (**A**, right). The following signs were observed during the trial: polyuria and diarrhea (**B**, upper left), cyanosis of the comb (**B**, upper right), fluffed plumage and general depression (**B**, lower left), and swollen eyelids and conjunctivitis (**B**, lower right). The following signs were detected *post mortem*: petechiae on serous membranes and in fatty tissue (**C**, left), ascites and fibrin exudation (**C**, center), and albuminous serous membranes (**C**, right). ALT: Altsteirer, BIE: Bielefelder, RAM: Ramelsloher, dpi: days post-infection

Clinical symptoms occurred later than in 6-week-old chickens and were first present in the BIE group at 5 dpi and latest in the RAM group at 7 dpi. Although differences between the breeds were not significant, RAM hens had the lowest clinical score and the highest survival rate. In contrast, ALT hens had the highest clinical score and the lowest survival rate (Figure 5A).

During the experiment, the following symptoms were observed: polyuria and diarrhea (Figure 5B upper left), cyanosis of the comb (Figure 5B upper right), fluffed plumage and general depression (Figure 5B lower left), and swollen eyelids and conjunctivitis (Figure 5B lower right). None of the infected chickens showed neurological or respiratory symptoms which are typical for HPAIV infection.

Owing to rather unspecific clinical symptoms, necropsy of each surviving animal with a subsequent gross pathology was performed at the end of the trial at 10 dpi. The following signs were detected *post mortem*: petechiae on serous membranes and in fatty tissue (Figure 5C left), ascites and fibrin exudation (Figure 5C center), and albuminous serous membranes (Figure 5C right). A quantitative categorization of these observations revealed that the majority of the infected chickens showed signs of peritonitis or salpingitis (ALT = 5/7 animals, BIE = 6/7 animals, RAM = 5/7 animals). However, the number of detected signs did not correlate with the severity of the disease (Additional file 4).

**Figure 6:**
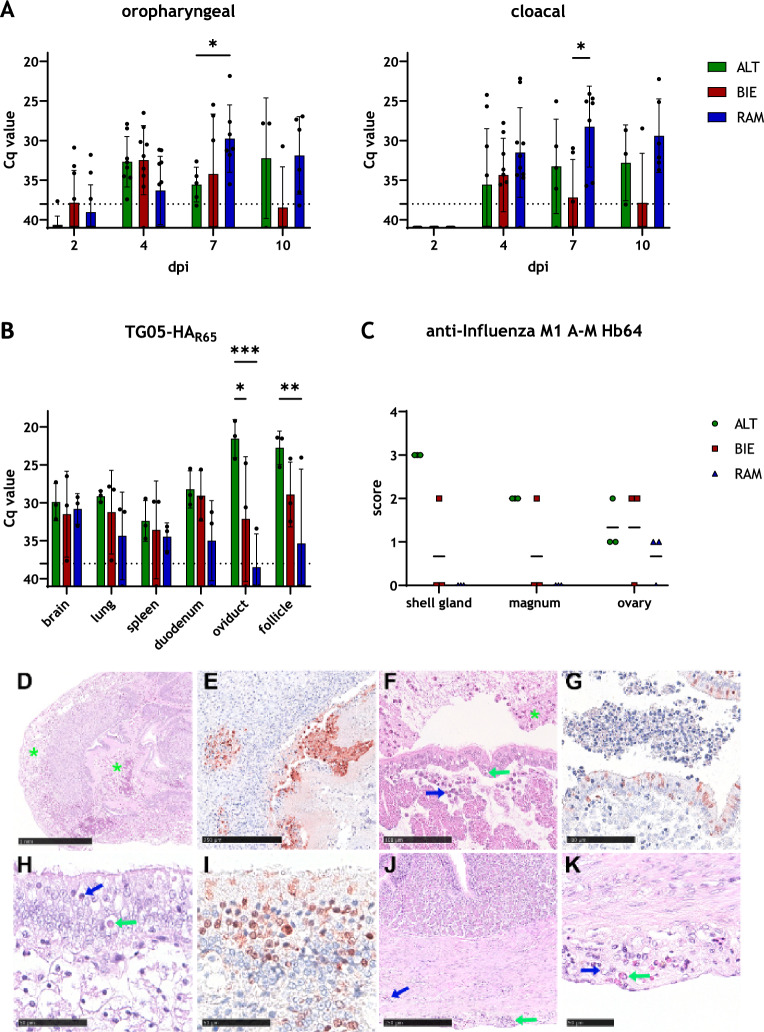
**Virus detection in TG05-HA**_**R65**_-infected laying hens. At 2, 4, 7, and 10 dpi, oropharyngeal (**A**, left) and cloacal (**A**, right) swab samples were analyzed by RT-PCR for virus shedding. Viral load in organ samples taken during necropsy at 4 dpi was analyzed by RT-PCR (**B**). Data are shown as mean with standard deviation and the dotted line marks the Cq threshold value of 38 (**A** and **B**). Microscopical scoring of influenza virus antigen-positive cells in various parts of the female reproductive tract. The mean is represented as a horizontal line (**C**). Representative sections of histopathologic evaluation and viral antigen detection by immunohistochemistry (IHC): ovary (BIE), oophoritis, with expansion of the ovary by abundant proteinaceous fluid and yolk (green asterisk), prominent edema and mixed cellular immune cell infiltrates, HE, bar 1 mm (**D**) and ovary, with influenza virus antigen detection in granulosa and theca cells 250 µm (**E**). Magnum (BIE), salpingitis, heterophilic, with mucosal single cell necrosis (green arrow), mainly heterophilic infiltrates (blue arrow), edema and intraluminal fibrillar, proteinaceous material, admixed with cellular debris (green asterisk), HE, bar 100 µm (**F**) and magnum, virus protein within mucosal surface epithelium, IHC, bar 100 µm (**G**). Shell gland (ALT), salpingitis, heterophilic, with mucosal single cell necrosis (green arrow), heterophilic transmigration (blue arrow), and edema, HE, bar 50 µm (**H**) and shell gland, influenza antigen labelling in mucosal surface epithelium and subjacent glands, IHC, bar 50 µm (**I**). Serosa of the magnum (BIE), serositis, acute, heterophilic, expanding the serosa (green arrow) and partially extending into the muscular layer (blue arrow), HE, bar 250 µm (**J**) and serosa of the magnum (detail of J), serositis, expansion of the serosa by heterophils (green arrow), macrophages (blue arrow) and edema, HE, bar 50 µm (**K**). Asterisks indicate statistical significance: (*) *P* < 0.05, (**) *P* < 0.01, (***) *P* < 0.001. ALT: Altsteirer, BIE: Bielefelder, RAM: Ramelsloher, dpi: days post-infection, IHC: immunohistochemistry

Analyzing viral load in organs from chickens that were used for necropsy at 4 dpi revealed differences between the breeds (Figure 6B). The highest viral load was found in the oviduct of ALT hens (mean Cq value (ALT) = 21.5). In ovarian follicles, high viral loads were detected in ALT and BIE hens (mean Cq value (ALT) = 22.7, mean Cq value (BIE) = 28.9). In contrast, RAM hens had significantly lower viral loads in both, the oviduct and ovarian follicles (mean Cq value oviduct (RAM) = 38.5, mean Cq value follicle (RAM) = 34.7). However, during necropsy, the ovaries of two of the three RAM hens were found to be inactive. Comparable viral loads with only minor, not significant differences were found in brain, lung, spleen, and duodenum. However, the highest viral loads were found in all organs of the ALT hens. Except for brain samples, it was noticeable that the viral load in RAM hens was consistently the lowest. To assess the influence of laying activity on infection-associated lesions and viral tissue tropism, histopathologic evaluation and viral antigen detection by immunohistochemistry was performed on lung, spleen, brain, oviduct (magnum, shell gland), and ovary (details and target cells given in Figures 6DK and Additional file 6). Confirming PCR-results, breed-specific differences were identified (Figure 6C). Viral antigen was detected consistently in ALT hens in the shell gland (score 3), magnum (score 2), and ovary (score 12). Limited labeling was recorded for two out of three BIE hens, including the lung (*n* = 1, score 1), shell gland (*n* = 1, score 2), magnum (*n *= 1, score 2), and ovary (*n* = 2, score 2). Most restricted viral antigen detection was found for RAM hens with labeling in the ovary in two out of three RAM hens (score 1). All remaining tissues were antigen negative. Several histologic lesions were interpreted to be associated with AIV infection based on antigen labeling within lesions (oviduct, ovary) or by absence of changes in mock-infected controls (lung, spleen, brain). No obvious differences were found between ALT, BIE, or RAM hens (details given in Additional file 6A). An acute salpingitis was identified (score 14) in the magnum and/or shell gland of all infected birds (Figure 6F, H). However, consistent with viral load data and antigen detection, ALT hens were most consistently affected reaching the highest severity score. In addition, affecting the oviduct, serositis (score 13) was a consistent finding in all breeds (Figure 6J). Only for one BIE hen, acute oophoritis was diagnosed (Figure 6D). However, it must be considered that oligofocal inflammation of the large, active ovary may have been missed in the evaluation of the relatively small sample. In addition, physiologic atresia and/or burst of large follicles was recorded for all birds. Although no viral antigen was detected in the brains of any of the breeds studied and the PCR data do not indicate breed-specific differences, only BIE chickens developed meningoencephalitis (score 2). Despite limited antigen detection, pneumonia with broncho-interstitial, predominantly heterophilic infiltrates was observed in all breeds but never exceeded score 2 (RAM 1/3; ALT 3/3, BIE 3/3). To assess the response of primary and tertiary lymphoid organs, the spleen and pulmonary bronchus-associated lymphoid tissue (BALT) were examined. Without significant differences, lymphoid cellularity was decreased, sometimes in association with acute lymphoid necrosis. In the spleen (score 2–3), almost all hens were affected, but in the BALT (score 1–2), all ALT and BIE hens but only one RAM animal was affected.

During the trial, the following data representing laying performance were recorded: the number of laid eggs, the average egg weight, and the presence of abnormal egg shell formation (Table 3). It could be seen that the laying performance decreased over the course of the trial. This decline was most evident in the ALT group: their laying performance was relatively high at the beginning, but decreased from 5 dpi onward until they stopped laying completely by 8 dpi. In the RAM group, the hens did not lay any eggs at 4 dpi; however, they continued laying 1 day later. From 8 dpi onward, their laying performance decreased again. Laying performance of the BIE group was comparatively unstable but a trend to declining could also be observed from 4 dpi onward. However, they were the only breed in which abnormal, shell-less eggs were found during the trial (at 1 dpi, 6 dpi, 9 dpi, and 10 dpi). The week before the infection experiment, these animals laid on average 0.51 eggs/hen (ALT and BIE chickens) or respectively 0.55 eggs/hen (RAM chickens). During the trial, no significant changes regarding egg weight could be detected (Table 3). However, it should be noted that the two noninfected animals (ALT: *n* = 1; BIE: *n* = 1), which were excluded from all other analyses and figures, could not be omitted from the assessment of egg production, as data on the number of eggs laid were not recorded individually per animal. Therefore, they remain included in this table.

**Table 3:** **Data representing laying performance during TG05-HA**_**R65**_ **infection**

Breed	dpi^d^	Amount eggs/hens	Mean egg weight	Shell-less eggs
ALT^a^	1	7/10	47.2	–
	2	6/10	48.7	–
	3	3/10	49.9	–
	4	7/10	47.7	–
	5	1/7	47.8	–
	6	3/7	47.2	–
	7	1/6	48.3	–
	8	0/6	–	–
	9	0/6	–	–
	10	0/4	–	–
BIE^b^	1	1/10	52.2	1
	2	4/10	49.1	–
	3	6/10	49.0	–
	4	0/10	–	–
	5	2/7	52.6	–
	6	1/7	51.9	1
	7	3/7	52.7	–
	8	1/5	54.2	–
	9	1/5	54.2	1
	10	1/5	52.4	1
RAM^c^	1	3/10	46.4	–
	2	5/10	48.1	–
	3	2/10	43.6	–
	4	0/10	–	–
	5	5/7	44.7	–
	6	6/7	44.3	–
	7	4/7	46.5	–
	8	0/6	–	–
	9	1/6	48.7	–
	10	1/6	48.2	–

^a^Altsteirer, ^b^Bielefelder, ^c^Ramelsloher, ^d^days post infection

To investigate whether laying hens under performance pressure have breed-specific differences in viral shedding, oropharyngeal and cloacal swabs were analyzed by RT-PCR (Figure 6A). The highest oropharyngeal virus shedding was seen in the RAM group at 7 dpi (mean Cq value (RAM) = 29.7). From 10 dpi onward, viral shedding decreased in both BIE and RAM groups; however, it increased in the ALT group. Compared with juvenile chickens (Figure 4A), higher levels of oropharyngeal virus shedding were generally observed in all breeds at 10 dpi (mean Cq value (ALT) = 32.2, mean (BIE) = 38.4, mean (RAM) = 31.9). The presence of virus particles was first detected in cloacal swab samples at 4 dpi. At all investigated time points, the highest titers were found in the RAM group.

Blood and lung samples were stained for flow cytometry and analyzed for immune cell infiltration. In contrast to 6-week-old chickens, an infiltration of the lung with CD8+ γδ T cells or CD8+ αβ T cells could not be observed in laying hens infected with recombinant TG05-HA_R65_. It was noticeable that ALT hens showed a decline in CD4+ T cells at 4 dpi. No further differences were observed between naive and infected hens in lung samples at 4 dpi (Additional file 5A). In blood samples, no breed-specific differences regarding the immune cell kinetics during infection were detected (Additional file 5B).

## Discussion

Although organic poultry farming places high demands on animal welfare and the environment, it also poses challenges such as an increased risk of infection. In particular, access to outdoor areas can facilitate contact with pathogens. In this context, the resilience of chicken breeds is becoming increasingly important. Breeds that are more resilient and better adapted to the conditions of organic farming with free-range husbandry systems could help reduce disease outbreaks [3, 38].

Cellular immune responses are crucial for defense against pathogens, in particular viruses, bacteria, and intracellular parasites [39–41]. T cells play a decisive role in the targeted recognition and elimination of infected cells [42–44].

In the present study, we investigated the cellular immune response to viral infection in three genetically distinct local chicken breeds: ALT, BIE, and RAM. To focus on the impact of host genetics on immunocompetence and viral resilience, we excluded all environmental and field conditions.

First, we challenged 6-week-old chickens with the highly pathogenic H7N1 A/Chicken/Italy/445/1999 strain. As expected, none of the chickens survived this infection, independently of their genetic background. Unexpectedly, no breed-specific differences in clinical signs and disease kinetics could be detected. The results of the present study are consistent with those observed by Abdelwhab et al. (2016) in infection experiments conducted on SPF White Leghorn chickens, in which all infected animals died from HPAIV H7N1 A/Chicken/Italy/445/1999 [45]. In contrast, Sánchez-González et al. (2020) were able to show that different chicken breeds have different susceptibilities to infection with the highly pathogenic H7N1 A/Chicken/Italy/5093/1999. Although it resulted in 100% mortality in some local Spain breeds and a mean mortality rate of 70%, only 40% of the infected SPF White Leghorn chickens died in this study [29].

In addition, the virus used in this study was isolated from an outbreak in Italy in 1999, emphasizing the importance of effective antiviral immunity in resilient poultry: at these times, measures to control animal diseases were not as strictly regulated as they are today. Consequently, an epidemic of LPAIV H7N1 in northern Italy led to virus circulation in flocks for months. This facilitated the direct evolution of an HPAIV from a low pathogenic precursor, causing over 413 outbreaks and resulting in the culling of 13 million birds [46, 47]. This indicates that LPAIV can also represent a risk to poultry, particularly in organic farming with contact to wild birds. Owing to the typically mild clinical signs and often asymptomatic courses, LPAIV frequently circulates undetected within flocks [13]. Therefore, it is crucial to utilize animals capable of autonomously controlling the infection through their own immune response.

To detect differences between the three chicken breeds used in the present study and compare the results with previously published data, the chickens were inoculated with the moderately pathogenic TG05-HA_R65_ model virus, which is known to result in a 70% survival rate in SPF White Leghorn chickens [14]. In another study by Blohm et al., infection experiments with four different chicken lines led to survival rates ranging from 40 to 100% [24]. In our study, we were able to show that infection with TG05-HA_R65_ in 6-week-old animals of our breeds resulted in different outcomes. While 86% of the RAM chickens survived, the survival rates were only 60% for BIE and 57% for ALT chickens, respectively. However, it should be noted that the mentioned studies by Bogs et al. and Blohm et al. involved infecting 2-week-old chickens, whereas here we used 6-week-old animals. Therefore, the results may not be directly comparable and it cannot be excluded that the minimal age difference plays a role. However, it is known that, unlike ducks and turkeys [48, 49], the age of chickens does not usually have a decisive influence on mortality and virus shedding after influenza virus infection [50]. Instead, host genetics and virus strain play a pivotal role in determining mortality and pathogenicity [51, 52]. This is consistent with our findings that mortality rates following influenza virus infection were comparable between 6-week-old chickens and 35-week-old laying hens within the respective breeds.

We demonstrated in 6-week-old chickens that, among all breeds, in both H7N1 and TG05-HA_R65_ infections, the lung was infiltrated by terminally differentiated CD8+ γδ T cells at 4 dpi. This supports the hypothesis that this cell subtype may play an important role in the early immune response [40]. In both infection experiments, we also demonstrated that cytotoxic CD8+ αβ T cells infiltrated the lungs in both BIE and RAM chickens. However, the increase was considerably more pronounced in the moderately pathogenic TG05-HA_R65_ infection. This infiltration of the lung likely contributes to viral clearance, as evidenced by oropharyngeal virus shedding: BIE and RAM chickens already tended to clear the virus by 7 dpi, and were excreting less virus than ALT chickens, which were shedding the highest levels at this time. There is a potential practical significance to this virus shedding with regard to transmission: the ALT chickens were the only ones to infect their sentinel animals. One of them died and most of them shed the virus both oropharyngeally and cloacally. In contrast, the ALT chickens tended to shed less virus cloacally at all analyzed time points. Similarly, the lowest viral load in the duodenum was found in ALT chickens. The actual reason for transmission exclusively occurring in the ALT group remains unclear. Since successful experimental virus propagation was confirmed in all three breeds, we can exclude the possibility that only the ALT group shed viable virus. However, transmission via the fecal-oral route appears to be less efficient compared with aerosol transmission which may explain the observed differences between breeds [53].

Studies with LPAIV have shown that viral clearance in oropharyngeal swab samples from infected chickens is mainly mediated by adaptive immune responses, particularly by the production of neutralizing antibodies against viral surface proteins HA and NA, as well as by the elimination of virus-infected cells by cytotoxic T cells. Although the innate immune response provides an initial barrier, complete viral clearance from mucosal membranes is due to the activation of specific immune mechanisms. Consequently, viral shedding decreases and eventually stops over the course of the infection [54].

In contrast, the infiltration of immune cells into the lung leads to a pronounced immune response and inflammatory reaction in chickens infected with avian influenza virus, particularly in cases of HPAIV [55]. In highly pathogenic H5N1 infections, massive infiltration of immune cells is associated with increased viral replication and severe lung damage, whereas milder infections (e.g. LPAIV H9N2) are associated with less inflammation [56]. Therefore, in the highly pathogenic H7N1 experiment of the present study, immune cell infiltration in the lung might have contributed to immunopathology and, consequently, the severity of the disease. However, Dai et al. (2023) identified infiltrating macrophages, rather than T cells, as the cause of inflammatory lung injury [56]. In contrast, Rebel et al. (2011) infected chickens with either LPAIV or HPAIV of the H7N1 subtype, and found similar levels of CD8α+ T cells, macrophages, and dendritic cells in the infected areas of the lungs of both experimental groups. However, the low pathogenic virus was only detected in some parts of the lung, whereas the highly pathogenic virus was found throughout the entire lung. Consequently, the authors concluded that the different localization of the viruses in the lungs might be responsible for their respective lethality [57]. Nevertheless, our study showed that infiltration of the lung with T cells was protective in the moderately pathogenic TG05-HA_R65_ infection, as evidenced by oropharyngeal clearance of the virus at 10 dpi, and by the absence of transmission to sentinel animals in the RAM and BIE groups.

Furthermore, we infected 35-week-old laying hens at their peak laying performance with moderately pathogenic TG05-HA_R65_ to assess the influence of laying activity on immunocompetence. In addition to genetics, laying activity influences immunocompetence, as both immune function and egg production are energy-demanding processes, and increased reproductive activity can impair immune function by competing for limited metabolic resources [32, 33]. An avian influenza vaccine study by Rudolf et al. showed that susceptibility to disease was highest when laying performance was at its maximum. Hens at the beginning and end of the laying period showed the lowest susceptibility [58].

Compared with the week before the infection experiment of the present study, a trend towards decreased laying performance was observed after infection. However, it is well recognized that laying performance in local breeds is generally more variable and less stable compared with commercial laying hybrids. Consequently, the direct assessment of the disease’s impact on laying performance is limited.

In contrast to the experiment conducted on 6-week-old chickens, in 35-week-old laying hens, no infiltration of cytotoxic immune cells into the lung could be detected at 4 dpi. This may explain why none of the 35-week-old laying hens, in contrast to 6-week-old chickens, were able to clear the virus completely from the respiratory tract, detectable by oropharyngeal swab samples at 10 dpi, towards the end of the experimental period. Generally, AIV infections in chickens initially affect the respiratory tract, with the lungs being an important target organ [56, 59]. However, HPAIV causes systemic infection affecting multiple organs, with the brain and the heart being the primary target organs [60, 61]. The lung plays a comparatively minor role in HPAIV replication, as demonstrated by our IHC data.

Interestingly, in laying hens, clinical symptoms occurred at later time points in the experiment and were less obviously pronounced compared with juvenile 6-week-old chickens. First symptoms occurred in the laying hen trial in the BIE breed at 5 dpi, in the ALT breed at 6 dpi, and in the RAM breed at 7 dpi; in juvenile chickens already at 3 dpi, among all breeds. Although host genetics and virus strain are the key determinants of virulence and pathogenicity, it is possible that the immune system of younger animals is juvenile without experienced vaccinations and infections [62–64]. Although direct studies on classical in the course of life developed ‘bystander immunity’, as observed in mammals, are rare in chickens, there is evidence to suggest that immune stimulation (e.g. through vaccines or previous infections) can enhance the general immune response to combat nonspecific pathogens. On the other hand, so-called “trained immunity” has been more extensively researched in chickens. This involves nonspecific immune activation following contact with immunostimulants, and can be considered the functional equivalent of bystander immunity. In this process, immune cells undergo epigenetic and metabolic changes that enable them to respond faster and more robustly to subsequent infections, including those caused by pathogens distinct from the initial trigger. For instance, probiotics and β-glucans can improve not only the specific immune response, but also general resistance to various bacterial and viral infections [65–67]. Consequently, younger chickens may be more susceptible to viral infections. Moreover, throughout life, various factors (e.g. nutrition, housing, environmental and social stress) act as immune modulators, contributing to the development and maturation of a differentiated immune system [68–71]. In addition, the symptoms in the laying hens were less pronounced and rather nonspecific, such as reduced general condition, feed intake and laying activity, and calmer behavior compared with the noninfected control group. Compared with TG05-HA_R65_-infected juvenile chickens, viral shedding in laying hens also appeared at later time points. While all infected juvenile chickens already had antibodies to NP of IAV by 7 dpi, at this time point, antibodies in laying hens were detected in only one individual per breed. Furthermore, the hens seemed more difficult to inoculate, as no virus replication could be detected in some individuals. Therefore, it can be concluded that all animals could possibly survive the infection if they did not have preexisting lesions in the oviduct due to their laying activity, which may favor egg yolk peritonitis [72]. Egg yolk peritonitis or salpingitis were revealed in the majority of the animals by gross pathology carried out on all animals that died during the infection experiment as well as on all surviving animals at the end of the trial. Similarly, Rudolf et al. (2010) observed a comparable clinical manifestation in a 2-year longitudinal field study in which an inactivated LPAIV H5N2-based vaccine was tested. Some of the animals challenged with HPAIV H5N1 showed fibrinous salpingitis or fibrinous and egg yolk-containing material in the peritoneum, regardless of whether they were vaccinated in advance or not. According to the authors, the AIV infection favored the development of an *Escherichia coli*-driven egg yolk peritonitis, suspecting that infection of ovarian cells increased the risk of the egg yolk becoming more fragile or rupturing [58]. Towards the end of the laying period, laying hens often exhibit ruptures in the ovarian vessels or follicles, as well as egg yolk peritonitis, owing to their age and laying performance. This could be confirmed by histology.

The entry of egg yolk into the abdominal cavity provides an optimal environment for bacterial proliferation, particularly for pathogens such as *Escherichia coli*. This bacterial colonization exacerbates preexisting lesions and tissue damage and facilitates ascending infections through the oviduct [72, 73]. Egg yolk peritonitis has also been observed in other LPAIV infections and outbreaks [74–77]. Bonfante et al. (2018) identified the infundibulum as the region of the oviduct most susceptible to infection [74]. In contrast, in the present study, the highest levels of viral antigen were detected in the shell gland of ALT hens. However, the ovaries were the only region in which the virus was detected across all breeds.

Overall, these findings demonstrate that older chickens display immune responses that could restrict viral replication. However, laying activity appears to promote the development of AIV-induced superinfections. Therefore, preventative measures are necessary, either through vaccination, as is already practiced in some countries, or with a well-adapted hygiene management system to minimize the risk of ascending bacterial secondary infections. Notably, there is evidence that salpingitis occurs more frequently in poultry kept in conventional housing systems than in free-range husbandry [78]. Thus, ecological farming may offer an advantage over conventional poultry farming in terms of animal health.

In conclusion, we demonstrated breed-specific differences in the antiviral immunocompetence between the three local chicken breeds ALT, BIE, and RAM. Besides viral pathogenicity, genetic make-up determines resilience to viral infections and age determines disease dynamics. We detected genetically determined differences in the immune response of juvenile chickens that were no longer detectable in adult laying hens under performance pressure. In addition, laying activity results in a different clinical manifestation compared with juvenile animals with inactive ovaries.

## Supplementary Information

**Additional file** **1.** **Additional flow cytometry data of lung T cells.** T cell subpopulations in the lung of H7N1-infected chickens compared to non-infected control animals (**A**) and in the lung of TG05-HA_R65_-infected chickens compared to non-infected control animals (**B**). Data are shown as mean with standard deviation. Asterisks indicate statistical significance: (*) *P* < 0.05, (**) *P* < 0.01, (***) *P* < 0.001. ALT: Altsteirer, BIE: Bielefelder, RAM: Ramelsloher, dpi: days post-infection.

**Additional file** **2. Flow cytometry data of blood T cells.** T cell subpopulations in the blood of H7N1-infected chickens compared to non-infected control animals (**A**) and in the blood of TG05-HA_R65_-infected chickens compared to non-infected control animals (**B**). Data are shown as mean with standard deviation. Asterisks indicate statistical significance: (*) *P* < 0.05, (**) *P* < 0.01. ALT: Altsteirer, BIE: Bielefelder, RAM: Ramelsloher, dpi: days post-infection.

**Additional file** **3. Seroconversion.** Plasma samples from naive animals at 0 dpi and from TG05-HA_R65_-infected chickens at 7 dpi as well as serum samples from surviving animals and sentinels at 10 dpi were analyzed by a competitive enzyme-linked immunosorbent assay for the detection of antibodies to Influenza A Virus nucleoprotein. Detection of antibodies in 6-week-old infected chickens and their sentinel animals (**A**). Detection of antibodies in 35-week-old infected laying hens (**B**). Data are shown as mean with standard deviation. *S/P* sample to positive rate, ALT: Altsteirer, BIE: Bielefelder, RAM: Ramelsloher, dpi: days post-infection.

**Additional file** **4. Data representing the number of signs for egg yolk peritonitis.** ALT: Altsteirer, BIE: Bielefelder, RAM: Ramelsloher.

**Additional file** **5. **Flow cytometry data of 35-week-old TG05-HA_R65_-infected laying hens. T cell subpopulations in the lung of infected laying hens compared to non-infected control animals (**A**) and in the blood of infected laying hens compared to non-infected control animals (**B**). Data are shown as mean with standard deviation. Asterisks indicate statistical significance: (*) *P* < 0.05, (**) *P* < 0.01, (***) *P* < 0.001. ALT: Altsteirer, BIE: Bielefelder, RAM: Ramelsloher, dpi: days post-infection.

**Additional file** **6. Raw data tables pathology.** Raw data for hematoxylin and eosin staining (**A**), the score defines as follows: 0 = no lesion; 1 = rare (< 5%), 1–3 foci, minimal; 2 = multifocal (6%–40%), > 3 foci, mild; 3 = coalescing (41%–80%), moderate; 4 = diffuse (> 80%), severe; ‘–‘ = not present on slide. Raw data for immunohistochemistry, the score defines as follows: 0 = no antigen; 1 = focal / oligofocal (< 5%), 1–3 foci; 2 = multifocal (6%–40%), > 3 foci; 4 = coalescing (41%–80%); 5 = diffuse (> 80%). ALT: Altsteirer, BIE: Bielefelder, RAM: Ramelsloher, BALT: bronchus-associated lymphoid tissue, PALS: periarteriolar lymphocyte sheaths (mainly T lymphocytes), *PELS* periellipsoid lymphocyte sheaths (mainly B-lymphocytes).

## Data Availability

The datasets used and analyzed during the current study are available from the corresponding author on reasonable request.
