# Supplementary figures and images for: Host genetics, lung T-cell immunity, and laying activity determine the disease outcome in avian influenza virus-infected chickens
Source: Vet Res. 2026 Jan 2;57:18. doi: 10.1186/s13567-025-01689-4 (PMC12849319; doi:10.1186/s13567-025-01689-4)

## Slide 1
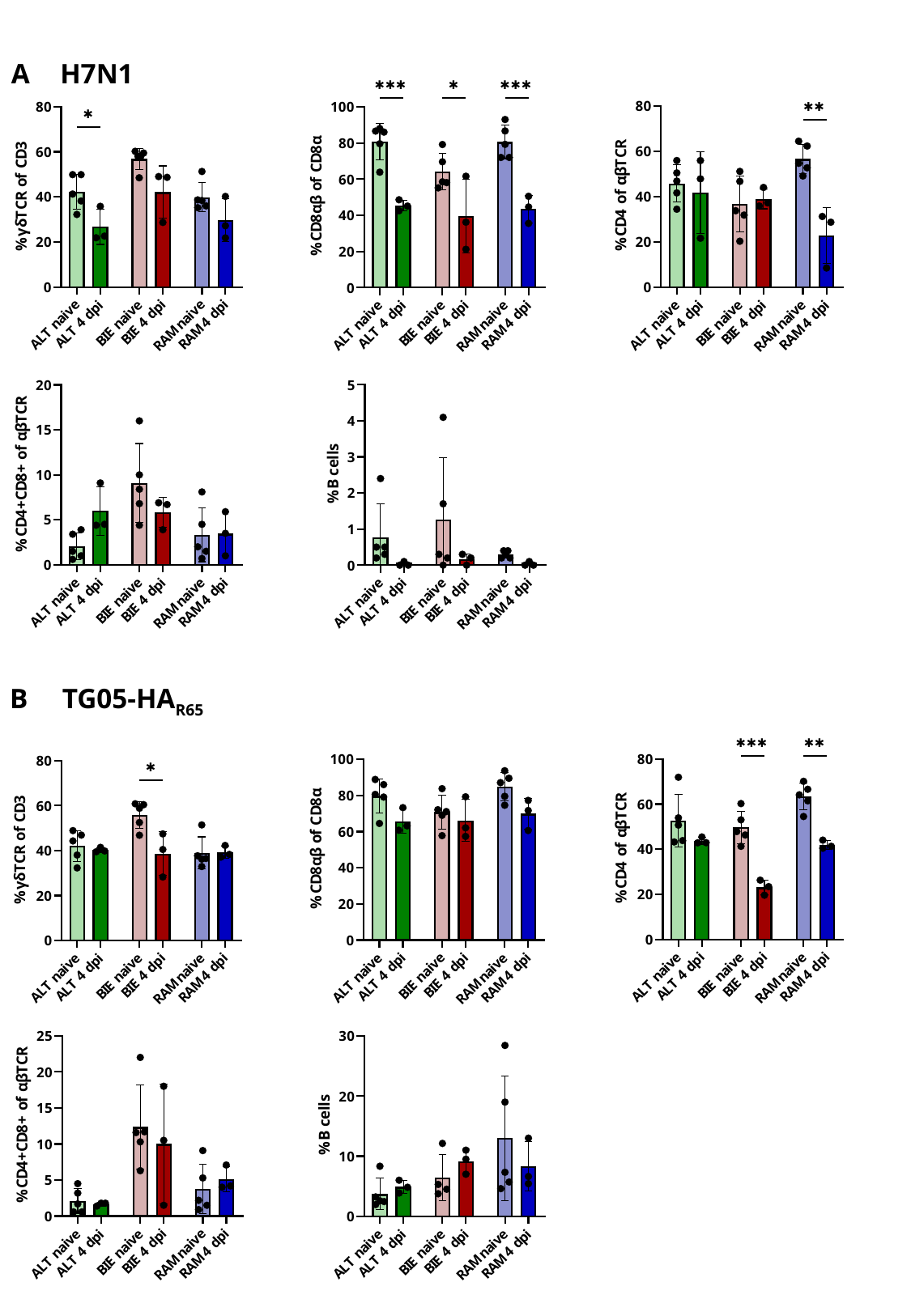

A
H7N1
B
TG05-HAR65

Supplement: Supplementary file 1 — Additional file 1. Additional flow cytometry data of lung T cells. T cell subpopulations in the lung of H7N1-infected chickens compared to non-infected control animals (A) and in the lung of TG05-HAR65-infected chickens compared to non-infected control animals (B). Data are shown as mean with standard deviation. Asterisks indicate statistical significance: (*) P < 0.05, (**) P < 0.01, (***) P < 0.001. ALT: Altsteirer, BIE: Bielefelder, RAM: Ramelsloher, dpi: days post-infection. [file 13567_2025_1689_MOESM1_ESM.pptx]

## Slide 1
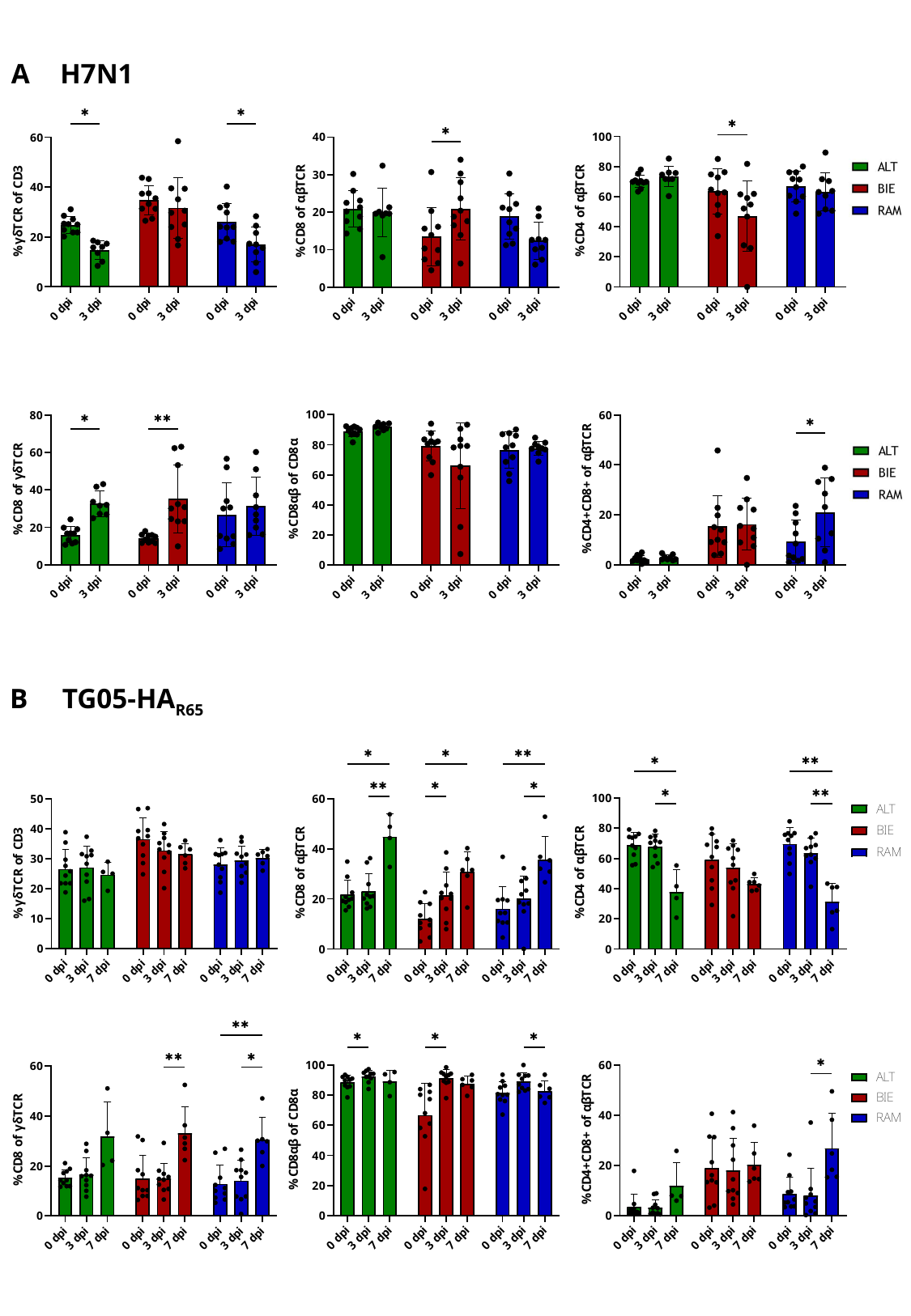

A
H7N1
B
TG05-HAR65

Supplement: Supplementary file 2 — Additional file 2. Flow cytometry data of blood T cells. T cell subpopulations in the blood of H7N1-infected chickens compared to non-infected control animals (A) and in the blood of TG05-HAR65-infected chickens compared to non-infected control animals (B). Data are shown as mean with standard deviation. Asterisks indicate statistical significance: (*) P < 0.05, (**) P < 0.01. ALT: Altsteirer, BIE: Bielefelder, RAM: Ramelsloher, dpi: days post-infection. [file 13567_2025_1689_MOESM2_ESM.pptx]

## Slide 1
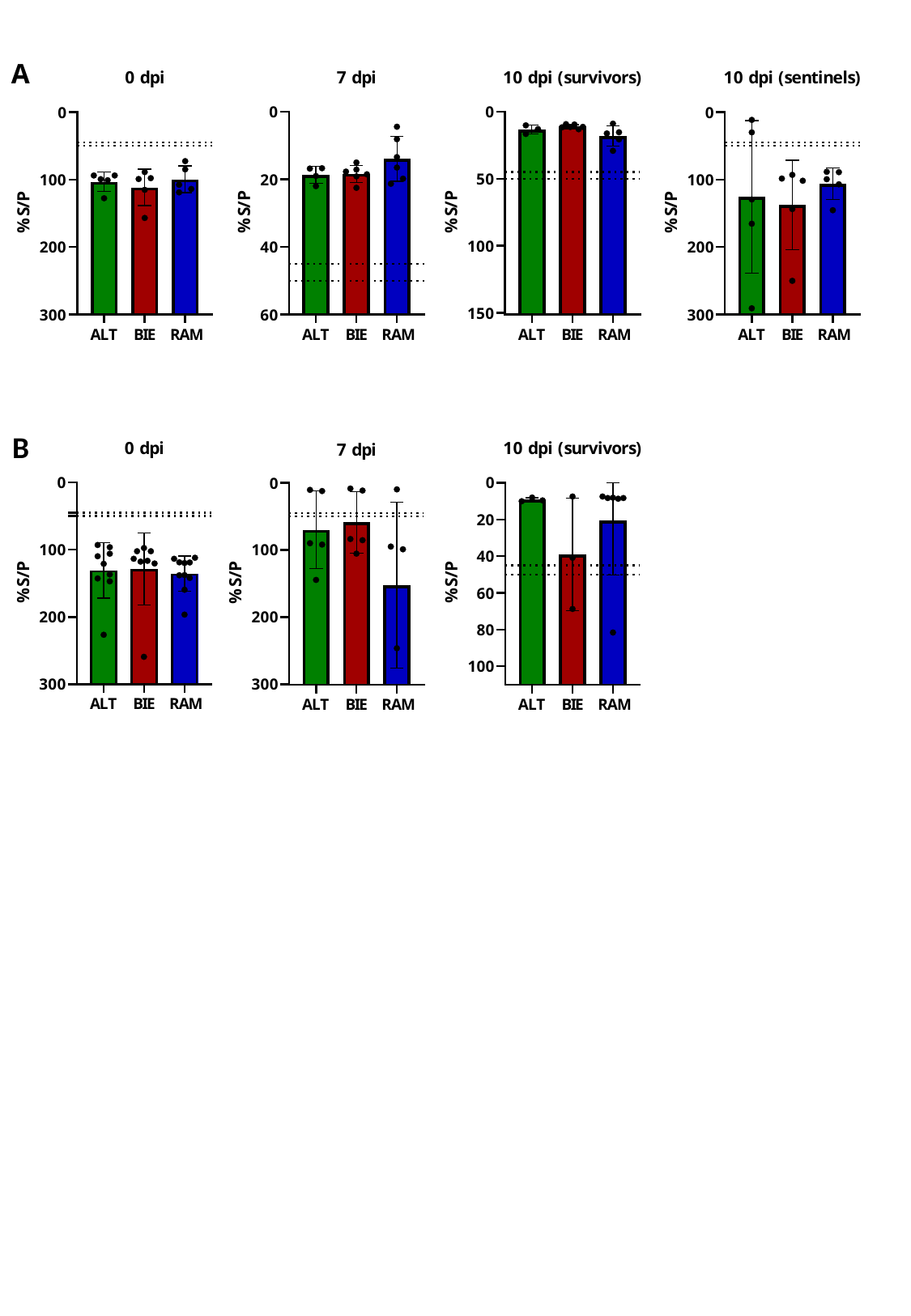

A
B

Supplement: Supplementary file 3 — Additional file 3. Seroconversion. Plasma samples from naive animals at 0 dpi and from TG05-HAR65-infected chickens at 7 dpi as well as serum samples from surviving animals and sentinels at 10 dpi were analyzed by a competitive enzyme-linked immunosorbent assay for the detection of antibodies to Influenza A Virus nucleoprotein. Detection of antibodies in 6-week-old infected chickens and their sentinel animals (A). Detection of antibodies in 35-week-old infected laying hens (B). Data are shown as mean with standard deviation. S/P sample to positive rate, ALT: Altsteirer, BIE: Bielefelder, RAM: Ramelsloher, dpi: days post-infection. [file 13567_2025_1689_MOESM3_ESM.pptx]

## Slide 1
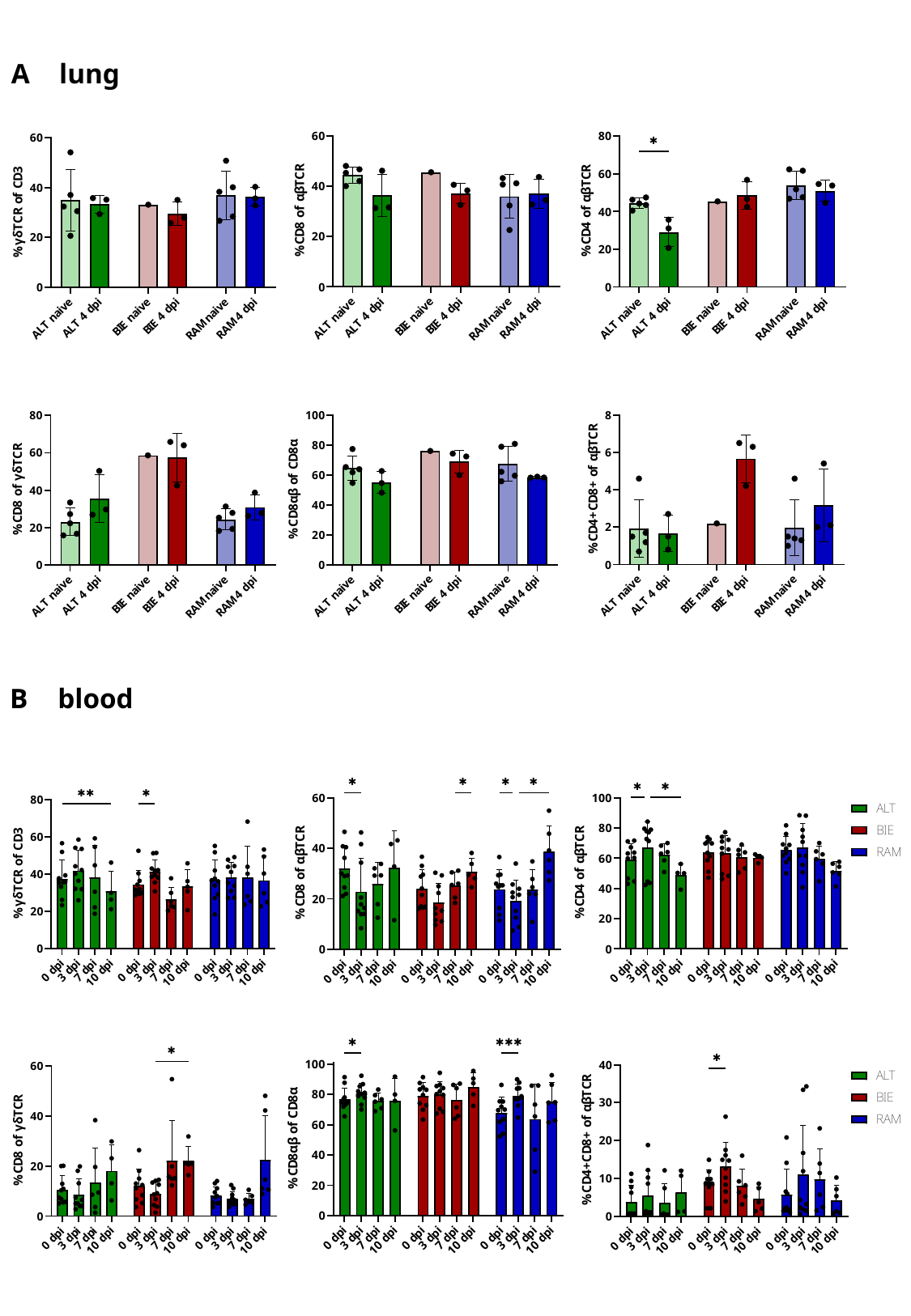

A
lung
B
blood

Supplement: Supplementary file 5 — Additional file 5. Flow cytometry data of 35-week-old TG05-HAR65-infected laying hens. T cell subpopulations in the lung of infected laying hens compared to non-infected control animals (A) and in the blood of infected laying hens compared to non-infected control animals (B). Data are shown as mean with standard deviation. Asterisks indicate statistical significance: (*) P < 0.05, (**) P < 0.01, (***) P < 0.001. ALT: Altsteirer, BIE: Bielefelder, RAM: Ramelsloher, dpi: days post-infection. [file 13567_2025_1689_MOESM5_ESM.pptx]

## Slide 1
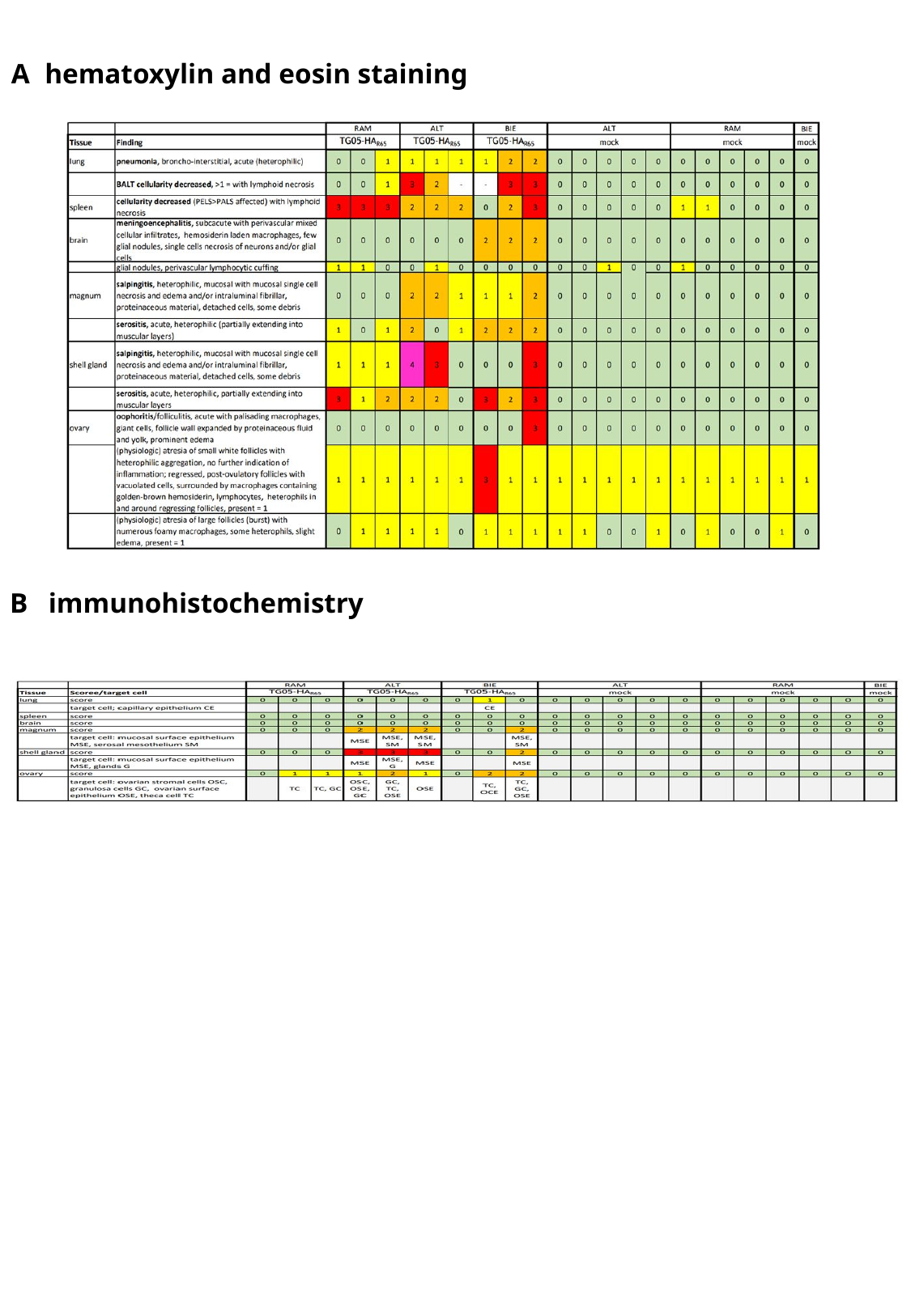

A
hematoxylin and eosin staining
B
immunohistochemistry

Supplement: Supplementary file 6 — Additional file 6. Raw data tables pathology. Raw data for hematoxylin and eosin staining (A), the score defines as follows: 0 = no lesion; 1 = rare (< 5%), 1–3 foci, minimal; 2 = multifocal (6%–40%), > 3 foci, mild; 3 = coalescing (41%–80%), moderate; 4 = diffuse (> 80%), severe; ‘–‘ = not present on slide. Raw data for immunohistochemistry, the score defines as follows: 0 = no antigen; 1 = focal / oligofocal (< 5%), 1–3 foci; 2 = multifocal (6%–40%), > 3 foci; 4 = coalescing (41%–80%); 5 = diffuse (> 80%). ALT: Altsteirer, BIE: Bielefelder, RAM: Ramelsloher, BALT: bronchus-associated lymphoid tissue, PALS: periarteriolar lymphocyte sheaths (mainly T lymphocytes), PELS periellipsoid lymphocyte sheaths (mainly B-lymphocytes). [file 13567_2025_1689_MOESM6_ESM.pptx]
