# Supplementary material for: Host genetics, lung T-cell immunity, and laying activity determine the disease outcome in avian influenza virus-infected chickens
Source: Vet Res. 2026 Jan 2;57:18. doi: 10.1186/s13567-025-01689-4 (PMC12849319; doi:10.1186/s13567-025-01689-4)
Supplement: Supplementary file 4 — Additional file 4. Data representing the number of signs for egg yolk peritonitis. ALT: Altsteirer, BIE: Bielefelder, RAM: Ramelsloher. [file 13567_2025_1689_MOESM4_ESM.pptx]

## Slide 1
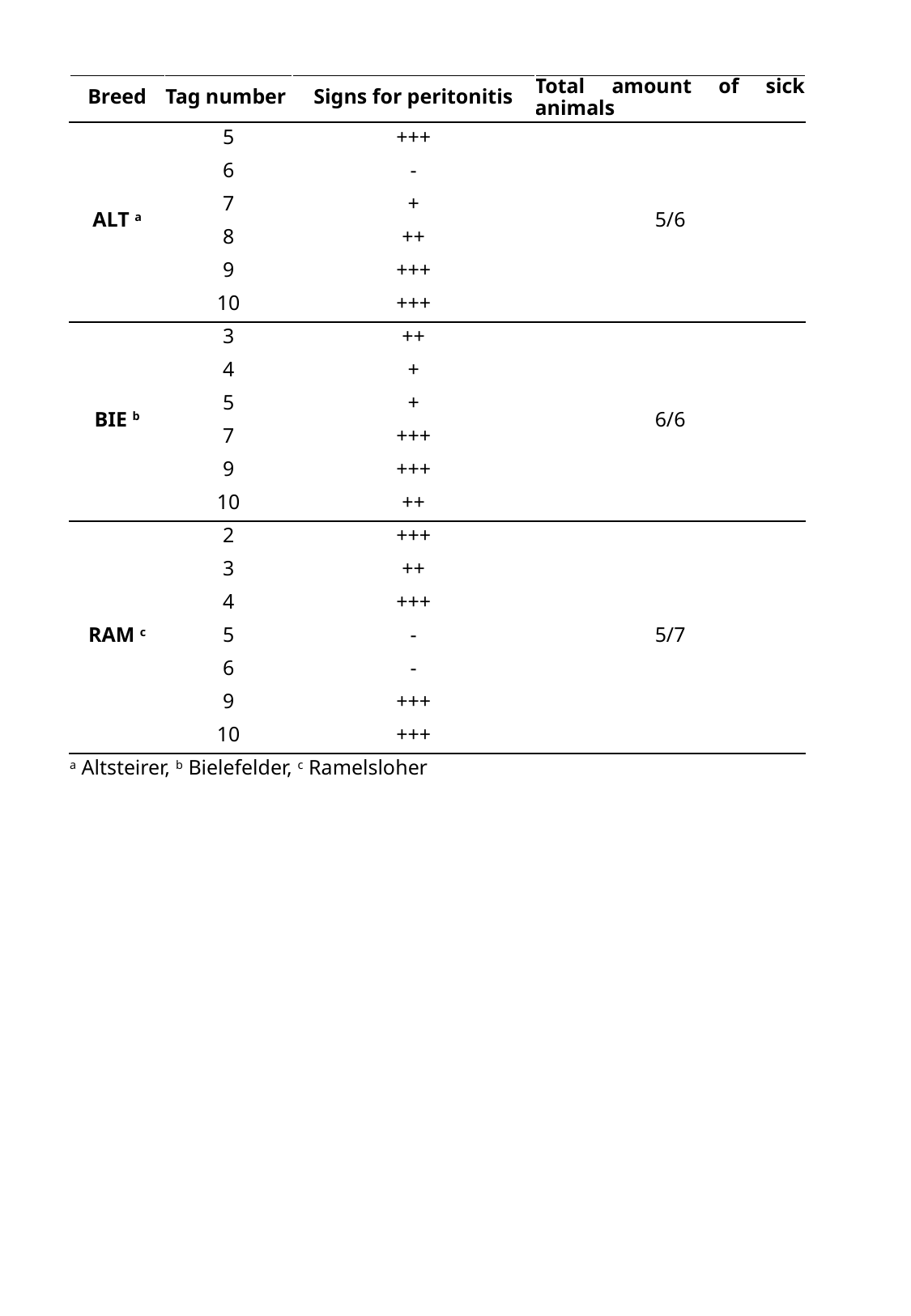

| Breed | Tag number | Signs for peritonitis | Total amount of sick animals |
| --- | --- | --- | --- |
| ALT a | 5 | +++ | 5/6 |
| | 6 | - | |
| | 7 | + | |
| | 8 | ++ | |
| | 9 | +++ | |
| | 10 | +++ | |
| BIE b | 3 | ++ | 6/6 |
| | 4 | + | |
| | 5 | + | |
| | 7 | +++ | |
| | 9 | +++ | |
| | 10 | ++ | |
| RAM c | 2 | +++ | 5/7 |
| | 3 | ++ | |
| | 4 | +++ | |
| | 5 | - | |
| | 6 | - | |
| | 9 | +++ | |
| | 10 | +++ | |
| a Altsteirer, b Bielefelder, c Ramelsloher | | | |
